# Development of Wheat With Hypoimmunogenic Gluten Obstructed by the Gene Editing Policy in Europe

**DOI:** 10.3389/fpls.2018.01523

**Published:** 2018-10-18

**Authors:** Aurélie Jouanin, Lesley Boyd, Richard G. F. Visser, Marinus J. M. Smulders

**Affiliations:** ^1^Plant Breeding, Wageningen University & Research, Wageningen, Netherlands; ^2^Genetics & Breeding Research, National Institute of Agricultural Botany, Cambridge, United Kingdom

**Keywords:** coeliac disease, mutation breeding, new plant breeding technique, public acceptance, innovation principle, GM regulation, genetic modification, risk assessment

## Abstract

Coeliac Disease (CD) is an auto-immune reaction to gluten in 1–2% of the human population. A gluten-free (GF) diet, excluding wheat, barley, and rye, is the only remedy. This diet is difficult to adhere to, partly because wheat gluten is added to many processed products for their viscoelastic properties. In addition, GF products are less healthy and expensive. Wheat products containing only hypoimmunogenic gluten proteins would be a desirable option. Various gluten peptides that trigger CD have been characterized. A single wheat variety contains around hundred gluten genes, producing proteins with varying numbers of epitopes. Gene editing using CRISPR/Cas9 can precisely remove or modify the DNA sequences coding for immunogenic peptides. Wheat with hypoimmunogenic gluten thus exemplifies the potential of gene editing for improving crops for human consumption where conventional breeding cannot succeed. We describe here, in relation to breeding hypoimmunogenic wheat varieties, the inconsistencies of applying GM regulation in Europe for gene-edited plants while mutation breeding-derived plants are exempted. We explain that healthy products derived from this new technology may become available in the United States, Canada, Argentina and other countries but not in Europe, because of strict regulation of unintended GM risk at the expense of reduction the existing immunogenicity risks of patients. We argue that regulation of gene-edited plants should be based on scientific evidence. Therefore, we strongly recommend implementing the innovation principle. Responsible Research and Innovation, involving stakeholders including CD patient societies in the development of gene-editing products, will enable progress toward healthy products and encourage public acceptance.

## Wheat Gluten and Coeliac Disease

Bread wheat (*Triticum aestivum*) is a staple crop consumed worldwide. The properties that make wheat flour suitable for bread-making are conferred by gluten, the glutenin and gliadin storage proteins present in the grain. High molecular weight (HMW) glutenins provide dough with elasticity, which is the most important property for bread quality, while gliadins provide viscosity (Shewry et al., 2009).

Wheat gliadins, and to a lesser extend low molecular weight (LMW) glutenins, carry immunogenic peptides that can cause Coeliac Disease (CD) in 1–2% of the human population (Fasano, 2006). CD leads to an inflammation of the small intestine, which affects nutrient absorption and causes diverse symptoms (Husby et al., 2012).

A gluten-free (GF) diet, excluding wheat, barley, and rye, is the only way CD patients can avoid symptoms. It is difficult to adhere to as wheat gluten is added to many food products (Atchison et al., 2010). Furthermore, current GF products are low in proteins and nutrients, high in salt and contain many additives to emulate the rheology of gluten-based dough (Caponio et al., 2008; Capriles and Arêas, 2014; Belz, 2016; Horstmann et al., 2016). Hence, healthier but safe products for CD patients are needed.

## Breeding Toward Hypoimmunogenic Wheat: a Complex Challenge

Breeding wheat without immunogenic epitopes (Gilissen et al., 2008, 2014) would be a definitive solution for CD patients (Shewry and Tatham, 2016). Developing “hypoimmunogenic gluten” wheat varieties that retain baking quality is, however, very challenging. Firstly, gluten proteins are encoded by five gene families containing many immunogenic epitopes. Within these families, α-gliadins on chromosomes 6 trigger CD strongly, followed by γ-gliadins, ω-gliadins, and LMW glutenins on chromosomes 1. Secondly, bread wheat is allohexaploid, with three sets of chromosomes referred to as genome A, B, and D. Each of these genomes contains all gluten gene families. As a result, a single bread wheat variety has a combination of gliadins and glutenins, some without any CD epitopes, others with one or more immunogenic epitopes (Van Herpen et al., 2006; Tye-Din et al., 2010; Salentijn et al., 2013; Ozuna et al., 2015). No cultivated wheat or wild relative has been identified that contains only CD safe gluten epitopes (Van den Broeck et al., 2010a,b). Consequently, conventional breeding alone cannot produce hypoimmunogenic varieties.

Gil-Humanes et al. (2010) used RNA interference to reduce the expression of the gliadin gene families by 97%, abolishing stimulation of T cells from CD patients while no major issues were reported regarding seed germination or dough quality (Gil-Humanes et al., 2014). Becker et al. (2012) reduced the expression of up to 20 α-gliadins, but other storage proteins became more abundant. As the transgenic RNAi construct remains in the wheat genome to silence the genes, these plants are subject to GM regulation, which in the EU is expensive, takes a long time, and has an uncertain outcome (Laursen, 2016). In practice this precludes investments in what initially will be a niche product.

Another approach is mutation breeding. Exposure to γ-irradiation has been used to randomly remove large regions of chromosomes in wheat, among which the gluten genes on chromosomes 1 and 6 (Van den Broeck et al., 2009). Selected mutations in separate plants can be combined by crossing and selecting, as was done for “ultra-low gluten” barley (Tanner et al., 2016). We screened a γ-irradiated population of variety Paragon (JIC, Norwich, United Kingdom) to identify relevant deletions in hexaploid bread wheat. Paradoxically, mutation breeding is regulated as conventional breeding based on a history of safe use, although it randomly alters or removes many other genes besides the intended ones.

## Crispr/Cas9 Editing of Gliadin Genes Toward Hypoimmunogenic Gluten

Gene editing (Baltes and Voytas, 2015), a prominent New Plant Breeding Technique (NPBT), can be used to develop wheat with hypoimmunogenic gluten (Jouanin et al., 2018; Sánchez-León et al., 2018). A Cas9 nuclease is directed by a guide RNA to a target region within the genome and generates a double strand break. Inaccurate DNA repair by the plant may result in mutations at the target site. As a pilot project, we focussed on mutating epitopes in α- and γ-gliadin genes – which are the most immunogenic – separately and simultaneously using CRISPR/Cas9. We transformed immature embryos of the bread wheat variety Fielder with constructs with Cas9 and multiplex guide RNA constructs, and regenerated plants. Due to the contiguity of the gliadin genes on the chromosome, gene copies located between two DNA breaks may be lost from the genome as well. Sánchez-León et al. (2018) successfully targeted α-gliadins with CRISPR/Cas9, generating small deletions. The Cas9 construct is to be out-crossed in subsequent generations (Schaeffer and Nakata, 2015; Sprink et al., 2015). Alternatively, Cas9 can be delivered through transient expression or as ribonucleoprotein (Zhang et al., 2016; Liang et al., 2017).

**FIGURE 1:**
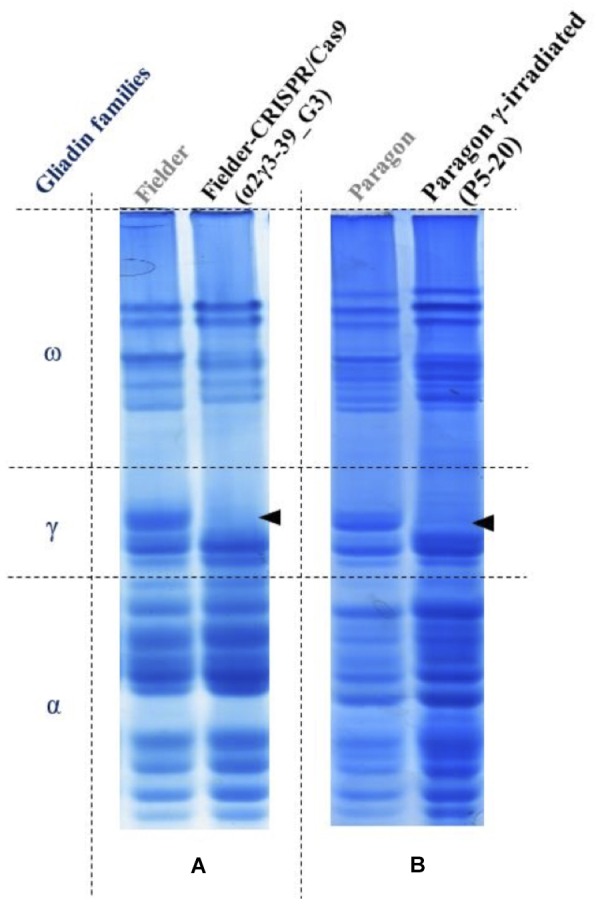
CRISPR/Cas9 gene editing **(A)** and γ-irradiation mutation breeding **(B)** generated similar changes in wheat γ-gliadin protein profiles from Jouanin et al. (2018). Lanes are Acid-PAGE gliadin profiles from control lines (on the left), a T1 Fielder grain, stably transformed with a CRISPR/Cas9 construct, and a M4 Paragon mutant line. Similar amounts of protein extract were loaded.

First, we tested grains of the plants produced for changes in gluten composition by acid-polyacrylamide gels, and determined the number of mutated or deleted gliadin genes using droplet digital PCR. Some γ-irradiated lines showed identical gliadin profile changes to gene-edited lines (**Figure 1**). Sequencing data enabled determining the type of mutations generated, while proteomics analysis can identify changes in amino acid composition of modified gliadin proteins. These data will enable predicting whether a mutation in an epitope decreases its immunogenicity, as crucial residues have been determined experimentally (Mitea et al., 2010) and the affinity of the human receptors has been fully characterized (Petersen et al., 2014, 2016).

Second, gluten from selected lines should be tested for immunogenicity and dough rheology. These tests are designed in collaboration with gastroenterologists, immunologists, food scientists, and CD patient associations. They comprise *in vitro* studies using epitope-specific T-cell clones isolated from CD patients (Anderson et al., 2000) and bread quality tests. Sánchez-León et al. (2018) made CRISPR/Cas9 mutant wheat lines with altered α-gliadin profiles and a reduction in immunogenicity, which retained acceptable dough quality.

As a third and final step, *in vivo* studies are needed where gluten from mutant grains would be given to voluntary CD patients to confirm hypoimmunogenicity. Then, hypoimmunogenic wheat will be ready to be cultivated in a separate production chain, carefully controlled from field to packaging to avoid contamination with regular wheat, barely or rye, similar to a GF oat chain (Smulders et al., 2018). It will likely be sold under a specific hypoimmunogenic gluten label.

## Gene-Edited Plant Varieties: Regulation, Safety, Acceptance and Policy in Europe

We describe here, in relation to hypoimmunogenic wheat, the inconsistencies of applying GM regulation for gene-edited plants in Europe while mutation breeding-derived plants are exempted. The EU regulation is based on the process used, not on the product generated, and follows the precautionary principle. Other countries have a product-based system (Canada) or a mixed product/process-based system (United States, Argentina).

### The Origin of GM Regulation for Gene Editing Plants in Europe

Competent Authorities of several EU countries, including the Swedish Board of Agriculture, as well as the European Food Safety Authority [EFSA], 2015) are in favor of adopting gene-edited products (Sprink et al., 2016a) with conventional breeding regulations or adapted regulations (Whelan and Lema, 2015). EFSA found a very low level of intended or unintended risks associated with site-directed mutated products (European Food Safety Authority [EFSA], 2012). Furthermore, the former Chief Scientific Advisor to the President of the European Commission (Simon, 2013) and the European Academies Science Advisory Council [EASAC], 2015) supported the regulation of gene editing plants as non-GM. However, the EC postponed a decision, mainly due to pressure from NGOs (Lawler, 2015). Recently, the European Court of Justice ruled that according to the text of the Directive 2001/18/EC, 2001. such products should be regulated as GM (European Court of Justice [ECJ], 2018a,b).

### Inconsistent Regulation of Mutated Plants in Europe

#### Random Versus Targeted Mutations

Mutation breeding deploys chemical mutagens or radiation. Because mutations occur randomly, large mutant populations must be screened to find a plant that contains the desired mutation, and each plant will contain many other mutations. These plants and products are considered as GM but exempted from GM regulation in most countries, including the EU (Directive 2001/18/EC, 2001. Annex 1B), due to a history of safe use and consumption since the 1930’s. Over 3200 commercial crop varieties have been produced using mutation breeding (Ahloowalia et al., 2004; Bado et al., 2015).

Gene editing uses a nuclease to generate a double-strand break at a desired target site in the genome, and plants are selected in which a mistake during repair led to a mutation of the target site. Off-targets may occur at a low frequency, much lower than in mutation breeding. In a product-based approach, the fact that plants obtained via gene editing are similar to those obtained using mutation breeding, means that they will follow the regulation of conventionally bred plants due to history of safe use (**Figure 1**). In contrast, in a process-based approach, as used by the EU, it has to go through the process of GM risk assessment.

#### Detrimental Effects on Costs and Opportunities

GM regulation of gene edited plants in Europe implies time-consuming (6 years) and costly ($35M) GM safety tests and administrative processes (McDougall, 2011), with uncertain outcome as the final permission is still a political decision. GM regulation erases the core advantages of gene editing as a quick, precise, and cheap method to develop high added-value plants to meet the needs of consumers and society.

**FIGURE 2:**
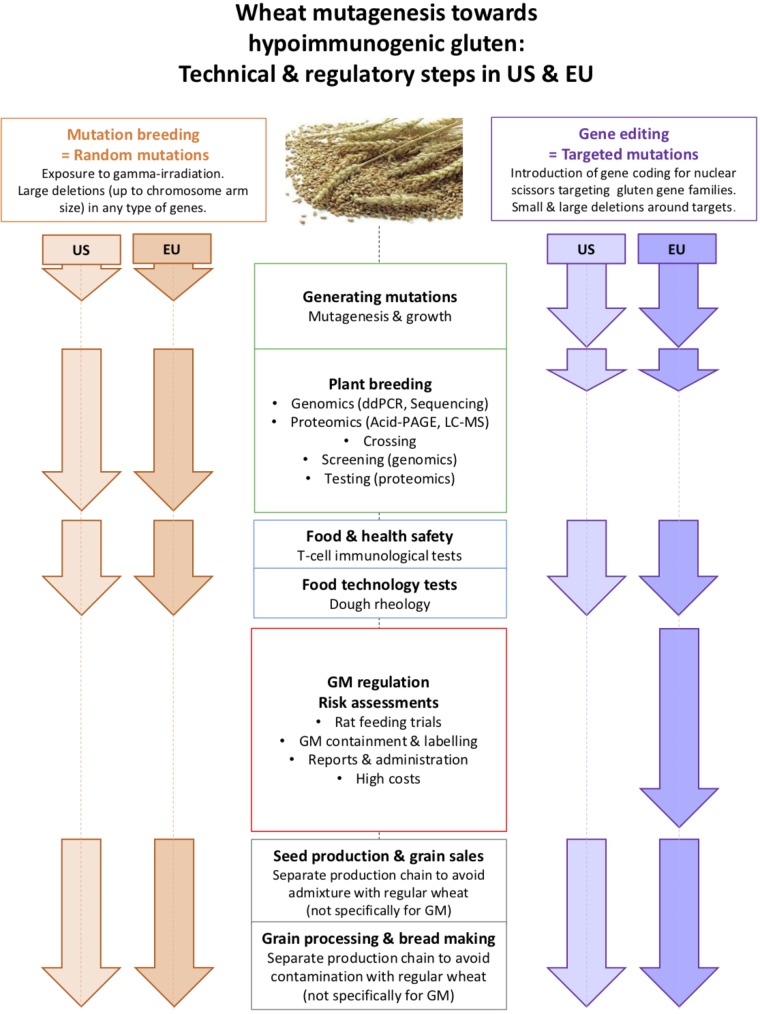
Comparison of the main technical and regulatory steps in the US and EU for breeding a hypoimmunogenic wheat variety using mutation breeding and gene editing.

In the United States, were both mutation breeding and gene editing are exempted from GM regulation, the latter will be preferred since it is more precise, faster, and versatile as it can produce homozygous mutations in several gene families simultaneously targeted (**Figure 2**). Consequently, European companies move their research facilities to the United States (Burger and Evans, 2018), and European researchers move to United States start-ups focusing on gene editing, such as Calyxt, which develops reduced-gluten wheat for the United States market. As hypoimmunogenic wheat will initially be a niche product, the costs of GM regulation will be too high for small and medium-sized companies in Europe. Thus, regulation of gene editing as GM will impede innovation, competitiveness, and access to healthier food in Europe.

#### Detection, Labeling and Effects on Trade

It is often impossible to distinguish products obtained using gene editing from those with mutation breeding or from ‘natural’, spontaneous mutations (Sprink et al., 2016b). The absence of distinctness will hamper control and labeling of gene editing-derived products, especially when it concerns material from outside Europe where gene-edited varieties are exempted from GM regulation. It represents a major issue. If Europe does not accept gene-edited products due to their lack of compliance with GM regulation applied in EU, this would block the import of any product that is not GM-labeled and tested. Indeed, any non-GM labeled product could potentially be produced with gene-editing, since there is no obligation of labeling gene-edited products in the United States. As a consequence, world trade could be disrupted (Cheyne, 2012).

Some gene-edited plants had similar targeted mutations as plants produced with γ-irradiation (**Figure 1**) and they cannot be distinguished by their gluten profile. In case of hypoimmunogenic wheat, a separate production chain is always required to avoid contamination with regular immunogenic wheat. The traceability is guaranteed, and products will be labeled as hypoimmunogenic, so it would be relatively easy to label them as derived from gene-edited wheat, even in the United States. For other products this will not be the case, as no separate production chain is necessary.

### Food Safety, Environmental Safety and Food Security Tests Under GM Regulation

For each new technology one undertakes a cost, benefit, and risk analysis. According to the (European Food Safety Authority [EFSA], 2012), the scientific facts gathered so far show no higher food safety risks of gene-edited plants than mutation breeding-derived plants that have a history of safe consumption. Furthermore, gene editing leads to plants with fewer off-targets modifications, making them at least as safe as conventionally bred ones (Lucht, 2015). This implies, from a risk assessment perspective, that gene-edited plants should be regulated as conventionally bred ones (European Plant Science Organisation [EPSO], 2015).

#### Food Safety Testing

The GM food safety risk assessment tests are related to the presence of foreign genes in the plants. These tests have not uncovered issues for over two decades (Swiss, 2012) and are not adapted for gene editing due to the absence of foreign genes introduced. In case of hypoimmunogenic wheat varieties, food safety issues will already thoroughly have been tested for coeliac patients, to ensure that epitope content is sufficiently low. However, to comply with the GM regulation for food safety, a rat feeding study has to be performed to test whether animals (that do not have CD) would develop unknown symptoms from eating hypoimmunogenic compared to regular wheat. On top of time, costs, and animal welfare issues, there is no relevance for these tests.

#### Environmental Safety Testing

Regarding environmental risks, under GM regulation, gene-edited plants have to follow strict containment rules. With regard to outcrossing, bread wheat is a self-pollinated species, and there are no wild populations. Outcrossing to other varieties would introduce hypoimmunogenic gluten, which is safer for human health, while bread quality would barely be affected. Gluten proteins are storage proteins in the grain and loss of gluten storage proteins did not lead to decreased fitness in ultra-low gluten barley (G.J. Tanner, CSIRO, Australia, Personal Communication).

#### Food Security

Considering food security, regulating gene editing as GM in Europe impedes the goals of increasing food production with fewer inputs (Ishii and Araki, 2016) for all types of agriculture, including integrated and organic farming (Andersen et al., 2015). As the economy of many developing countries relies on food exports to the EU, regulating gene editing as GM in the EU consequently has a negative impact on the availability of the technology for local markets in these countries, affecting their food security (Heap, 2013).

### Public Acceptance and Responsible Research and Innovation

The public needs to be better informed about new food technologies, to enable educated choices about food consumption. Scientists should contribute to this knowledge transfer and creation of awareness. However, the complexity of science often confuses people’s risk perception, decreasing their trust in scientific facts and increasing their fears, that they base on inaccurate information or wrong concepts from non-scientific sources (Lucht, 2015). This contributes to empower NGOs that influence the regulation-making process by claiming to protect consumers’ safety on no scientific grounds.

In a context where scientific communication has proven to be insufficient, the Responsible Research and Innovation initiative (RRI) (Owen et al., 2012) should be implemented as complementary approach. Targeted consumers should be asked for their interest in a potential product benefiting them and their trust in the methods used, in order to assess product acceptance prior its development, and they should remain involved during the whole process.

CD patients are the prime consumers for gene-edited hypoimmunogenic wheat. Following this RRI initiative, the idea of developing such a product has been discussed with CD patient associations early on. They understand the complexity of the challenge and appreciate the effort of scientists to develop a solution, even if the initial results are not perfect, as often when developing products concerning health issues (Schenk et al., 2011).

Gene-edited hypoimmunogenic wheat fits into a strongly growing market of GF food for coeliacs and other consumers (Sapone et al., 2012). In addition, it may contribute to preventing genetically predisposed children of developing CD, as quantity of exposure matters (Koning, 2012). Thus, there is a clear prospective gain in health which is held back in Europe by the GM regulation of gene editing. CD patients, relatives and others benefiting from gene-edited products should stand up and help the scientific community to convince politicians to adopt a science-based regulation of gene-edited plants and derived products.

### Policy Making: “Innovation Principle” Instead of “Precautionary Principle”

Considering the incoherence of applying GM regulation in EU to gene-edited products that may be identical to conventional varieties and anticipating its consequences in terms of food and environmental safety, food security, as well as associated economic issues, we strongly urge the EC to review its position on the matter. So far, “the precautionary principle” (European Commission [EC], 2000) is being applied solely, although technically this principle, meant as a provisional measure to avoid discernible risks based on scientific evidence, is not valid anymore considering the history of safe use of GM [no evidence of hazards for 20 years (Swiss, 2012)]. We argue that the “innovation principle” (European Political Strategy Centre [EPSC], 2016) should be used instead where relevant risk assessment would be designed on a case-per-case base, to enable benefiting of gene-edited products while complying with relevant risks management. This would constitute an appropriate regulation for the future of food security, healthy food, as well as protection of the environment and economy.

## Conclusion and Recommendations

Gene editing has made it possible to remove CD epitopes from wheat gluten. It is expected that in America, derived-products from such wheat will be on the market soon. Due to their absence of compliance with GM regulation, these products will remain illegal in the EU, as long as gene-edited products will be regulated as GM, following a process-based approach.

In addition, these niche products would not be developed in EU either due to the lack of profitability associated with expensive GM regulation tests and labeling. These GM tests, based on an precautionary principle, are required to detect unintended effects associated to transgenes, which are not present in the product.

We argue that, instead, gene-edited plants should be regulated as plants made with mutation breeding, on a product-based approach, and follow the innovation principle. This principle values benefits associated with the product while scientifically complying with trait-specific risk management. It should be part of a RRI involving targeted consumers as stakeholders, to ensure their acceptance throughout the gene-edited product development process.

Food safety, environmental safety, and food security in Europe will directly be affected by the regulation of gene editing as GM, and we expect politico-economic issues related to non-GM regulation of gene editing in other countries. Therefore, we strongly advise the EC to review its position on NPBT regulation by considering the present case and the regulatory advices provided.

## Author Contributions

AJ conceived this perspective and wrote the first version. MS adapted the manuscript. LB and RV introduced arguments and edited the text. AJ and MS edited the final version. All authors approved the final version.

## Conflict of Interest Statement

The authors declare that the research was conducted in the absence of any commercial or financial relationships that could be construed as a potential conflict of interest.
